# Dual-Function Conductive Copper Hollow Fibers for Microfiltration and Anti-biofouling in Electrochemical Membrane Bioreactors

**DOI:** 10.3389/fchem.2018.00445

**Published:** 2018-09-25

**Authors:** Defei Liu, Xin Chen, Bin Bian, Zhiping Lai, Yue Situ

**Affiliations:** ^1^School of Environment and Chemical Engineering, Foshan University, Foshan, China; ^2^School of Chemistry and Chemical Engineering, South China University of Technology, Guangzhou, China; ^3^Environmental Science and Engineering, King Abdullah University of Science and Technology, Thuwal, Saudi Arabia

**Keywords:** copper hollow fiber, anti-biofouling, microfiltration, electrochemical membrane bioreactor, water recovery

## Abstract

Membrane bioreactors (MBRs) with polymeric/ceramic microfiltration (MF) membranes have been commonly used for wastewater treatment today. However, membrane biofouling often results in a dramatically-reduced service life of MF membranes, which limits the application of this technology. In this study, Cu hollow fiber membranes (Cu-HFMs) with low resistivity (104.8–309.8 nΩ·m) and anti-biofouling properties were successfully synthesized. Further analysis demonstrated that Cu-HFMs reduced at 625°C achieved the bimodal pore size distribution of ~1 μm and a porosity of 46%, which enable high N_2_ permeance (1.56 × 10^−5^ mol/m^2^ s pa) and pure water flux (5812 LMH/bar). The Cu-HFMs were further applied as the conductive cathodes, as well as MF membranes, in the electrochemical membrane bioreactor (EMBR) system that was enriched with domestic wastewater at an applied voltage of 0.9 V. Excellent permeate quality (Total suspended solids (TSS) = 11 mg/L) was achieved at a flux of 9.47 LMH after Cu-HFM filtration, with relatively stable transmembrane pressure (TMP) and low Cu^2+^ dissolvability (<25 μg/L). The anti-biofouling over time was demonstrated by SEM characterization of the rare biofilm formation on the Cu-HFM cathode surface. By using Cu-HFMs in EMBR systems, an effective strategy to control the membrane biofouling is developed in this study.

## Introduction

Water scarcity is becoming a global issue and severely hindering the development of rural areas. Over the past decades, industrialization in developing countries has achieved huge advances, but introduced large amounts of wastewater at the same time, which conversely intensifies the global water scarcity. Wastewater reclamation and reuse are thus essential to avoid water shortage and environment pollution (Hou et al., 2016). Bio-electrochemical systems (BES), such as microbial fuel cells and microbial electrolysis cells, have emerged in recent years to directly degrade organic matters in wastewater and produce electricity or clean biofuel (e.g., CH_4_ or H_2_) at the cathode (Lovley, 2012; Katuri et al., 2018). Though BES could remove more than 80% Chemical Oxygen Demand (COD) in the synthetic wastewater, the reclaimed effluent still contains substantial amounts of biomass or suspended solids (Zhang and He, 2013). In order to reject the biomass/suspended solids and improve the effluent quality, membrane filtration was integrated with BES to jointly work as electrochemical membrane bioreactors (EMBRs), which keep the strengths of both sides and thus have great potential for wastewater treatment (Ozgun et al., 2013; Yuan and He, 2015).

Polymeric membranes, made from nylon (Wang et al., 2011), PVDF (Li and He, 2015), or cellulose (Kim et al., 2015), have been proposed as excellent ultra/micro filters for the wastewater treatment in EMBR systems. However, due to the electrical insulation of these polymeric membranes, additional metal/carbon electrodes are required in these EMBR systems for electrogenic bacterial colonization and COD removal (Yuan and He, 2015). To further reduce the cost of materials and the footprint of bioreactors, novel conductive membrane materials, such as carbon-coated stainless-steel mesh (Akamatsu et al., 2010), nickel hollow fibers (Katuri et al., 2014) and carbon felt (Zhang et al., 2014), have been recently reported and attracted lots of attention due to their dual functions as membranes and cathode electrodes in EMBRs. Excellent effluent quality after filtration and COD removal via microbial electrochemical degradation were achieved simultaneously in these EMBR systems, with good amount of energy recovery into biofuels (Katuri et al., 2014, 2018; Yuan and He, 2015). Nevertheless, long-term and sustainable operation of EMBRs with these dual-function membrane cathodes remains a challenge, particularly due to membrane bio-fouling issues. Both external fouling (i.e., the growth of biofilm) and internal fouling (i.e., adsorption of humic acids and deposition of salts) could prompt the membrane clogging in the long term, which are difficult to avoid in the wastewater treatment (Ho et al., 2017). Besides, severe bio-fouling in these EMBR systems will significantly reduce the water filtration flux, leading to the higher transmembrane pressure and energy demands for water filtration (Katuri et al., 2014; Werner et al., 2016), and thus result in the instability of electrochemical performances (Malaeb et al., 2013; Myung et al., 2018).

Recently, several *in-situ* membrane cleaning strategies have been proposed to alleviate the membrane bio-fouling of the dual-functional membrane cathodes in EMBR systems (Huang et al., 2015; Katuri et al., 2018). Huang et al. utilized the carbon-based flat sheet as the membrane electrode and applied high electrical field (higher than 2 V/cm) in the EMBR system, which effectively mitigated the membrane bio-fouling problem by introducing H_2_O_2_ oxidative compounds at the surface of conductive membrane cathodes (Huang et al., 2015). Werner et al. (Werner et al., 2016) constructed the EMBR system with the graphene-coated nickel hollow fiber cathode and successfully reduced the bio-clogging via hydrogen bubbling produced at the cathode surface at an applied potential as low as 0.7 V. Both approaches have effectively inhibited bio-clogging of the membrane pores and postponed the membrane cleaning cycles. However, the follow-up chemical and physical cleaning is yet required for thick biofilm removal on the cathode after long-term EMBR operation. To truly inhibit the excessive bacterial growth on the membrane, further modifications are needed for membrane electrode materials to obtain surface antibiotic properties, which may increase the cost for the electrode preparation. One possible alternative for the membrane fouling control in EMBR systems is to use copper membranes as EMBR electrodes (Ho et al., 2017). This approach has been demonstrated to work efficiently by Myung et al. (Myung et al., 2018), where copper mesh, serving as one kind of fouling-resistant cathodes in microbial fuel cells (MFCs), experienced far less bio-clogging than stainless steel mesh. Besides, several studies have reported the excellent conductivity of copper electrodes in BES compared with carbon and other metal materials (Baudler et al., 2015, 2017; Bian et al., 2018), which could help enhance the current density and reduce the ohmic loss. However, copper-based membrane electrodes and their anti-biofouling performances in the EMBR system were rarely discussed. Hence, it's worthwhile to develop dual-function and conductive copper membrane electrode to test their anti-fouling performance in EMBR systems.

In this study, the highly conductive Cu hollow fiber membranes (Cu-HFMs) were prepared by phase-inversion processes, followed by the oxidizing-reductive sintering. The preparation conditions for Cu-HFMs with optimal pore size and filtration properties were extensively investigated. Cu-HFMs were applied as conductive cathodes, as well as microfiltration (MF) membranes, in the EMBR system. To characterize the performances of the Cu-HFM cathode in EMBR, COD removal efficiency, biomass rejection and effluent quality were studied. Bio-fouling control and copper corrosion were analyzed to make sure no copper dissolved into the media and bacterial colonization was inhibited on the Cu-HFM surface.

## Experimental methods

### Fabrication of Cu-HFMs

The Cu-HFM precursors were first prepared from the Cu solution via a phase-inversion spinning process and then used to fabricate the Cu-HFMs via oxidizing-reductive sintering. The detailed synthesis procedures were described in Supplementary S1.1-S1.4.

### Cu-HFM characterization

The morphology and microstructures of the Cu-HFMs were characterized by scanning electron microscope (SEM, Quanta 600). X-ray powder diffraction (XRD, Rigaku D/Max 2500 V/PC) with a sweeping rate of 5.0° min^−1^ was utilized to analyze the structures of Cu-HFMs at different preparation stages. Thermogravimetric analysis (TGA) was performed using Q600 SDT thermal analysis machine (TA Instruments, USA), ranging from 50 to 700°C with a ramping rate of 10°C min^−1^. The pore size distribution of Cu-HFMs was measured via a mercury porosimeter (Micromeritics, AutoPoreIV9500) with the mercury pressure from 1.50 psi to 33,000 psi. To study the permeation properties of different Cu-HFMs (15 cm long), inert N_2_ gas permeation across the membranes was carried out as well as DI water flux permeation with a cross-flow filtration system. The apparatus was operated at a feed velocity of 3.0 m/s and a transmembrane pressure (TMP) of 1 bars before the measurement of the permeate flux. The mechanical strength and the electrical resistivity ρ of the Cu-HFMs were also measured via a three-point bending instrument (cross head speed 0.5 mm/min, Instron Model 5544) (Rahman et al., 2015) and four point method (PPMS-Dynacool, Quantum Design) (Dharmasena and Wadley, 2002), respectively. All the measurements were conducted at room temperature.

### Electrochemical membrane reactor construction and operation

**Scheme 1:**
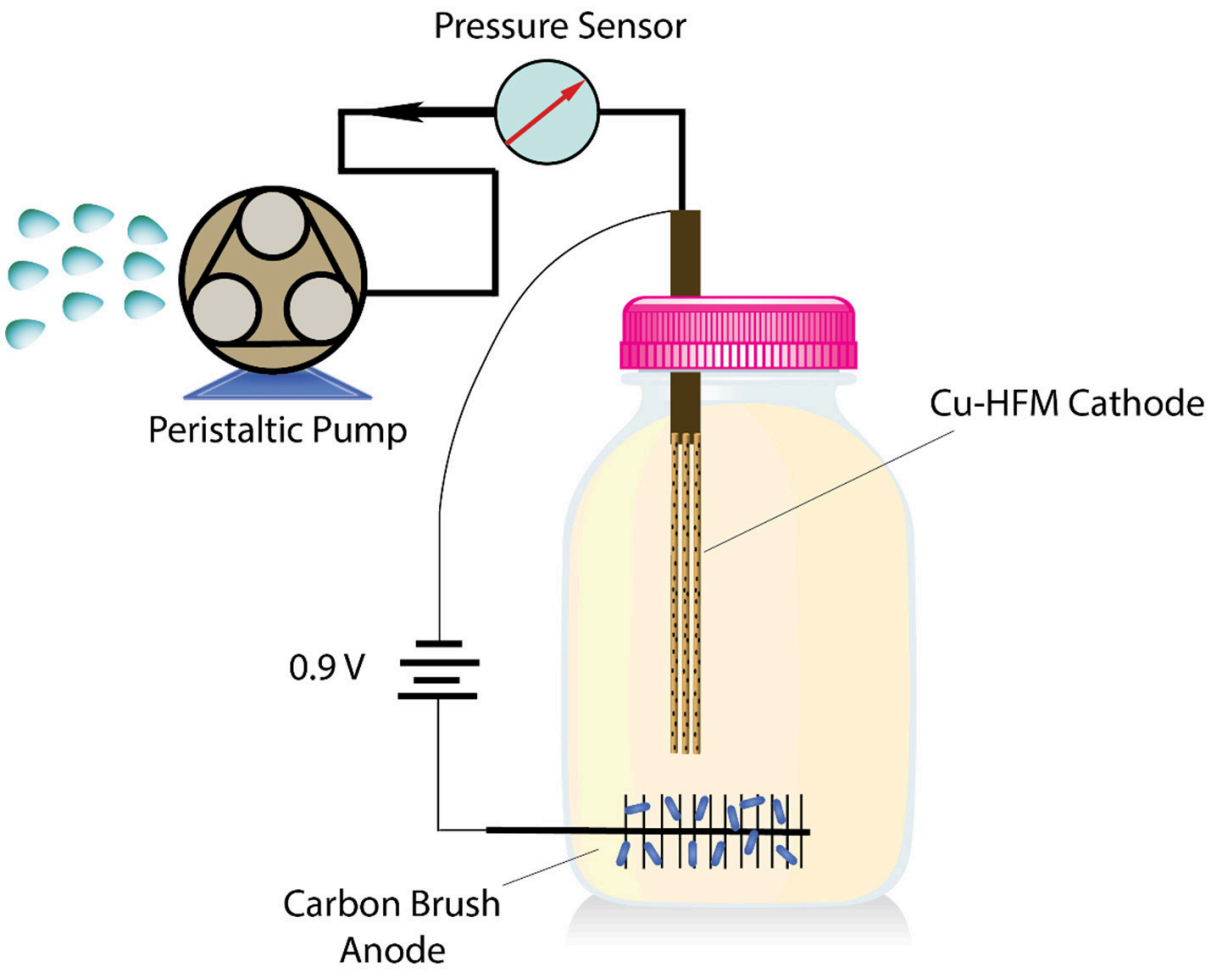
Schematic diagram of the EMBR system used in this study.

The Cu-HFMs prepared above served as the membrane cathodes (projected surface area of 4.43 cm^2^) in the anaerobic EMBR (Scheme 1), which was sparged with N_2_ for 20 min before operation. One end of the Cu-HFM cathode was inserted into a silicone tubing, which was connected to a peristaltic pump (Masterflex L/S, Cole-Parmer), and the joint point was sealed at with epoxy. The other end of the Cu-HFM cathode was also sealed with a drop of epoxy to form the dead-end membrane filter. A voltage of 0.9 V was applied between the carbon fiber brush anode (8 × ø 3 cm, ZOLTEK) and the Cu-HFM cathode, both submerged in 250 ml of synthetic wastewater with acetate (0.32 g COD/L) in the EMBR, to prevent the possible copper corrosion and accumulate bacterial biofilm on the anode for COD removal. After bacterial enrichment for 1 month with anoxic digest sludge (10% v/v, KAUST Wastewater Treatment Plant, Thuwal, KSA) and the peak voltage reached steady state, no sludge was added to the EMBR chamber except the synthetic wastewater containing acetate. Before we changed the batch at 20% peak current density, the COD concentration in the media was detected with Hach COD kit to characterize the EMBR COD removal efficiency, as well as the transmembrane pressure (TMP) detected by a pressure sensor (68075-32, Cole-Parmer) when ~50% of the treated media was filtered through the Cu-HFM cathode at a permeate flux of 9.47 L/m^2^/h (LMH). (Katuri et al., 2014; Werner et al., 2016) UV-Vis absorbance spectrometry at a wavelength of 600 nm and total suspended solids (TSS) were utilized to characterize the permeate quality after Cu-HFM filtration in the EMBR, following the methods reported in literature (Albertson, 2007; Katal and Pahlavanzadeh, 2011; Hassanshahian et al., 2013). The copper ion concentration in the EMBR system was detected with ICP-MS (7500 Series, Agilent Technologies) to identify whether copper corrosion occurred during the EMBR operation. Biofouling of Cu-HFM cathode was characterized by SEM, and energy dispersive spectroscopy (EDS) was utilized to analyze the element changes on Cu-HFM cathode surface after 57 days of operation. The operation details could be found in Supplementary S1.5.

## Results and discussion

The preparation of Cu-HFMs from discrete copper particles via the established phase-inversion process involves the spinning of hollow fiber precursors (HFPs) and high-temperature oxidizing-reductive sintering. The robust HFPs made from copper particles sufficiently bind by Polysulfone (PSF) could extend up to 25 cm long without breaking as shown in Figure S1a. Moreover, all the copper particles were evenly wrapped by the polymeric matrix (Figure S1b), which served as uniform gaps between copper particles and was burnt to form porous surface during the sintering process. In order to obtain the excellent conductivity and the optimal porous structures of Cu-HFMs for microfiltration, the oxidizing-reductive sintering process was extensively studied.

**Figure 1:**
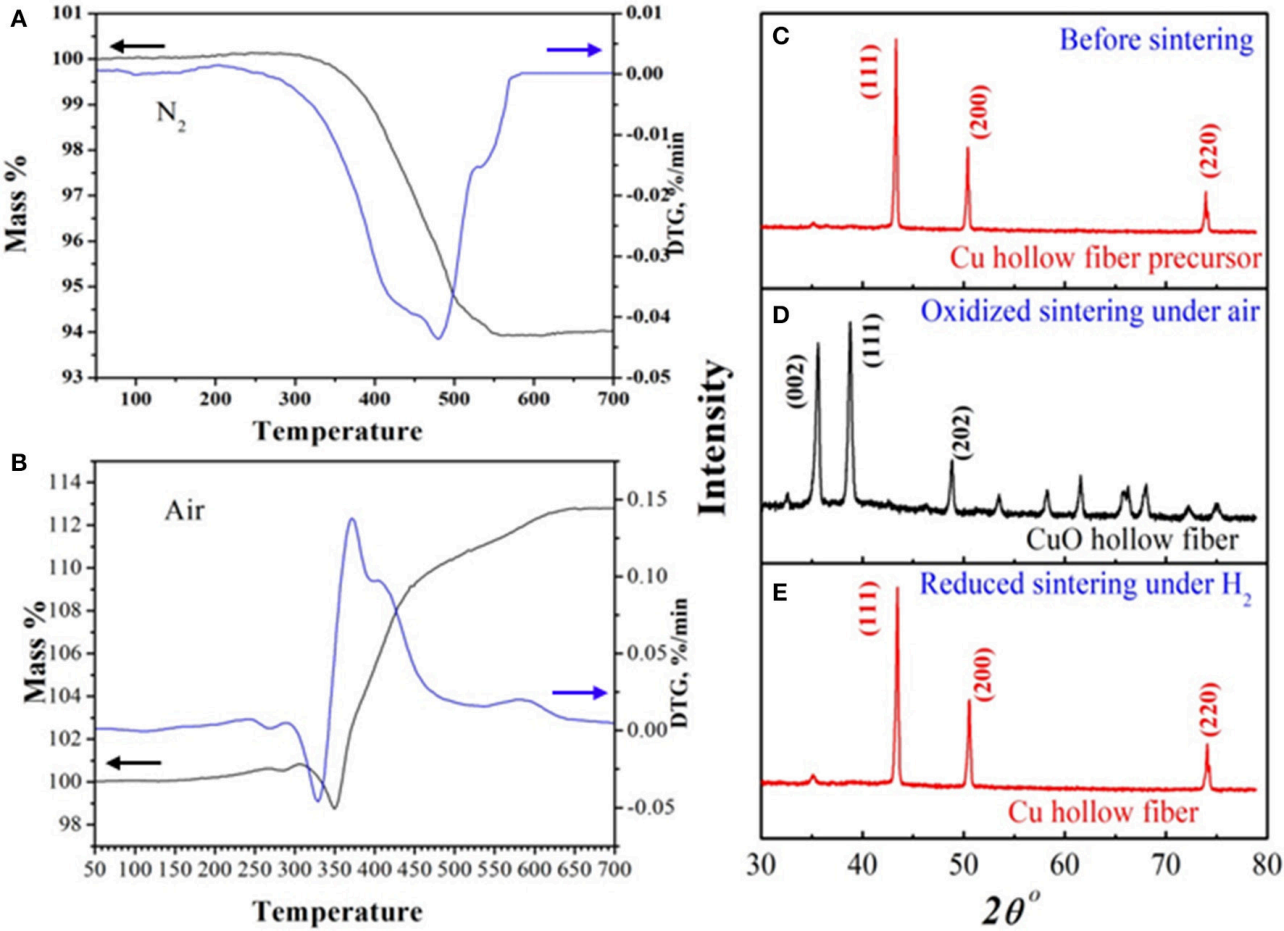
TGA curves of the Cu HFMs precursor in **(A)** N2 and **(B)** air atmospheres; XRD patterns of the **(C)** Cu hollow fiber precursor, **(D)** CuO hollow fiber and **(E)** Cu hollow fiber.

To investigate the oxidizing-reductive sintering condition of the Cu-HFM preparation, the TG curves of the Cu HFPs in air and N_2_ atmosphere were measured, respectively, from 50°C to 700°C. No obvious weight loss was observed below 300°C, while a sharp decline in sample weight started from 300° to 550°C in nitrogen, as shown in Figure 1A. The total weight loss was <6 %, which was significantly lower than the polymer content in Cu-HFPs (~11%). This weight loss could be attributed to the pyrolysis of the polymer binder, which forms carbonaceous particles and blocks the pores of Cu-HFMs (Li et al., 2015). To completely remove the polymer binders, air flow was selected for the TG combustion during the Cu-HFP sintering process (Figure 1B). It's obvious to see the sharp weight loss of the samples starting from 300°C, which illustrates the polymer decomposition in the precursors. However, the sample weight notably rebounded at around 350°C and kept increasing until the temperature reached 600°C, exhibiting totally different weight-temperature trend in N_2_ atmosphere. This rebound commonly results from the oxidation of copper particles in air starting from 350°C (Zhu et al., 2005). The weight of the HFPs finally remained constant from 600°C to 700°C, achieving a weight increase up to 13% after sintering in air. The optimal temperature for complete oxidation of Cu HFPs is expected to reach at least 600°C.

The X-ray diffraction (XRD) pattern was used to detect the crystal structures of the synthesized Cu HFPs (Figure 1C), CuO HFs sintered at 600°C in air (Figure 1D) and final Cu HFs reduced at 400°C with H_2_ (Figure 1E). The Cu HFPs and Cu HFs have the same diffraction peak locations (43.3, 50.4, and 74.0°), suggesting the same cubic structure of metal Cu (JCPDS: 04-0836). However, the diffraction peaks at *ca*. 35.5, 38.7, and 48.6° were indexed for CuO HFs, corresponding to the monoclinic structure of CuO (JCPDS: 80-1917). When we further investigated the data, it clearly demonstrated that the Cu metal particles in the HFPs could be completely converted to copper oxide at 600°C in air, which was subsequently reduced to Cu-HFMs with H_2_.

Figures S1c,d illustrate the microstructures of the CuO-HF cross-section after 600°C sintering in air for 3 h, suggesting the finger-like porous structures could be maintained even after burning out the polymer binders. Li et al. (Li et al., 2015) reported that different reducing temperatures played a significant role in determining the microstructures of metal hollow fibers and thus affected the physical properties, such as pore sizes, conductivity and filtration performances. To reduce CuO to Cu HFs and obtain excellent filtration and conductivity, the reducing temperatures were set at 400°C, 500°C, 625°C and 700°C, respectively, in H_2_ atmosphere. The reducing temperature was set higher than 400°C to make sure the polymer binders were totally removed, and the copper particles were partially fused to form strong bonding between each other, which leads to relatively high mechanical strength.

**Figure 2:**
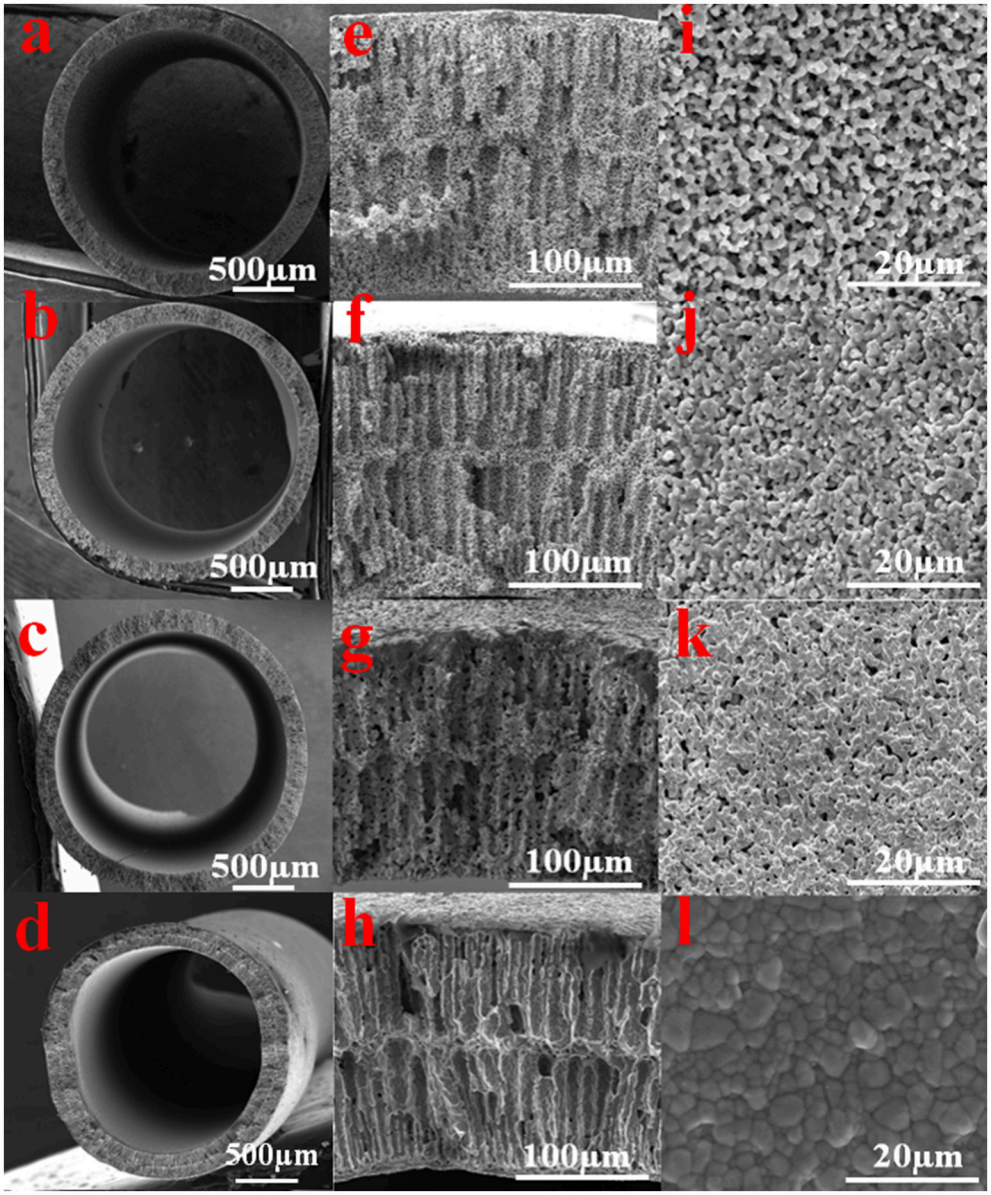
SEM images of the Cu-HFMs reductive sintered for 3 h in H_2_ atmosphere at different temperatures **(a,e,i)** 400°C; **(b,f,j)** 500°C; **(c,g,k)** 625°C and **(d,h,l)** 700°C. **(a–h)** for cross sectional; **(i–l)** for outer surface.

Figures 2a–l display the cross-section and outer surface of the Cu-HFMs reduced at different temperatures, respectively. Long finger-like structures are clearly presented in Figures 2e–h near the outer and inner walls of all the hollow fibers, while sponge-like structures are observed in the middle of the hollow fiber walls. The different appearance of the fiber structures across the HF wall could be attributed to the rapid solidification of the polymer binders at both inner and outer fiber walls with water coagulant flowing through in the phase-inversion process (Li et al., 2015). Furthermore, the surface micropores (~3 μm) formed due to the removal of polymer binders and incomplete sintered Cu particles were well preserved on the hollow fiber outer surface after reduction sintering at 400°C (Figures 2e,i). However, the discrete copper particles reduced at 500°C could hardly be recognized on the hollow fiber surface (Figure 2j), even though the porous structures were still evident at the cross-section (Figure 2f). Since the copper particles on the surface usually undergo a softening and bonding process during the CuO-HF reduction period, increasing the reducing temperature prompts more aggressive fusion and bonding of copper particles, which results in the particle growth and the decrease of the Cu-HF porosity (Li et al., 2015). This phenomenon was confirmed in Figures 2g,k, as the pore size on the hollow fiber surface shrank to 1 μm and the individual copper particles were no longer distinguishable after reduction at 625°C. When the temperature was increased to 700°C, no obvious micropores could be observed (Figure 2l) and the copper particles were tightly bonded with visible boundaries on the compact HF surface. The particle growth during the sintering could be clearly witnessed since most grains were larger than the original particle size (~1 μm). The typical finger-like porous structures, however, still exist at the cross-section of Cu HFs (Figure 2h).

**Figure 3:**
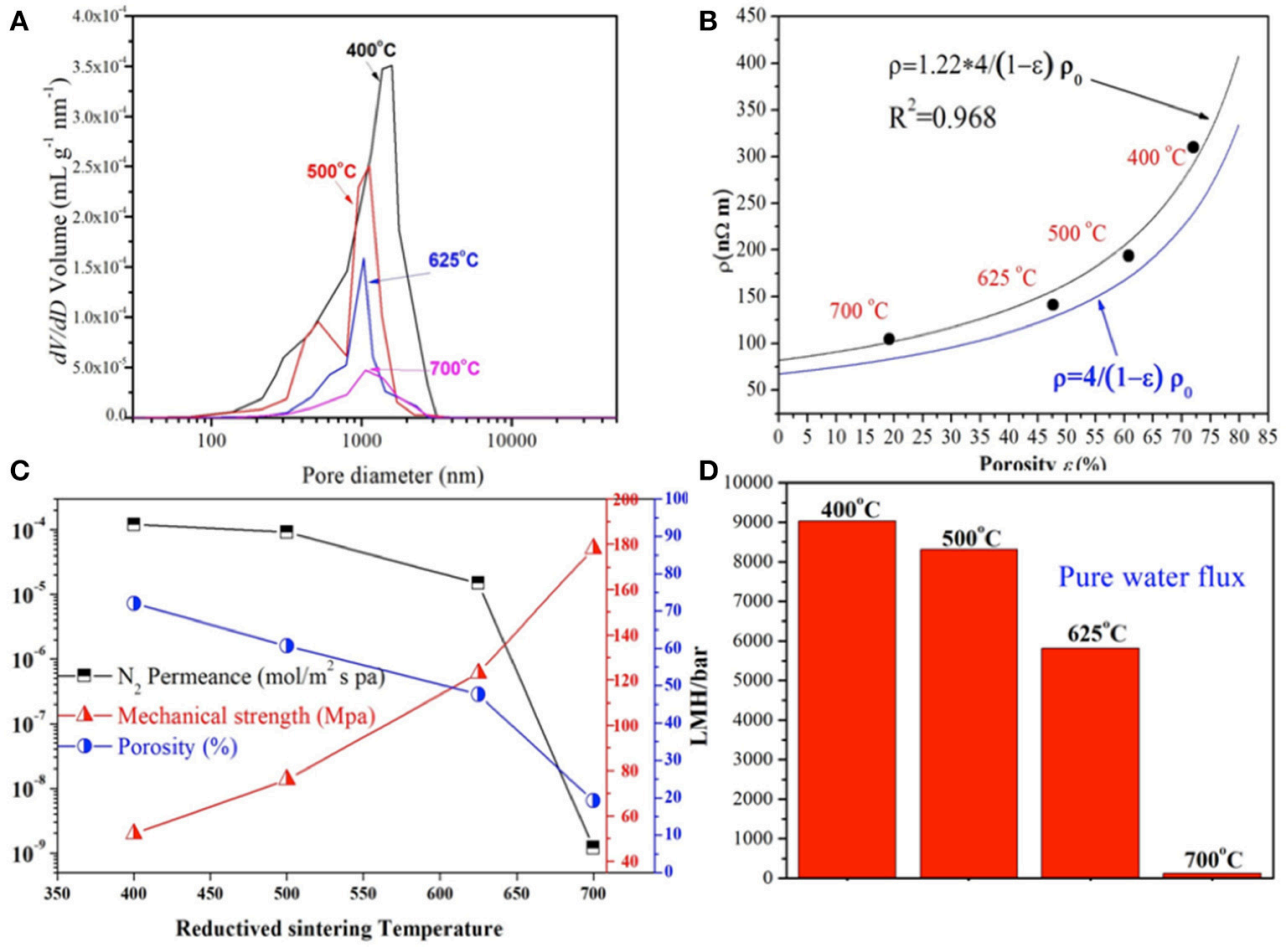
Pore size distribution of the Cu-HFMs reduced sintering at different temperatures **(A)**; Measured resistivity of Cu-HFMs with different porosities **(B)**; Porosity, N_2_ gas permeance, mechanical strength **(C)** and pure water permeation data **(D)** of the Cu-HFMs prepared at different temperatures.

To evaluate the properties of different Cu HFs, the pore size distribution at different reductive-sintering temperatures was determined by the mercury porosimetry analyzer, as depicted in Figure 3A. The fibers reduced at 400°C had a pore size distribution from 200 nm to 3 μm. The smaller pores (200 nm ~ 1 μm) were induced by the interstices between the un-sintered copper particles, while larger voids (>1 μm) formed at the location where the polymer binders were burned out in the oxidized-sintering process (Luiten-Olieman et al., 2011). With the increase of the reductive-sintering temperature, the percentage of the smaller pores induced by copper particles interstices, began to decline due to the excellent melting and bonding of most copper particles. Also the larger ones (>1 μm) shrank in size, narrowing down the pore size distribution to ~ 1 μm, which was suitable for microfiltration in MBRs. However, the peak height of the larger pores (~1 μm) was significantly reduced when the temperature reached 700°C. This could be attributed to the high sintering temperature, leading to the micropore blockage by melt copper particles and reduced pore density. It's noteworthy that the large micropores lie in the range of about 1 μm, which was probably determined by the original particle size of the copper powders (~ 1 μm) and hardly changed with the sintering temperature.

As we know, copper is one of the most conductive metal materials. However, the conductivity of most metals will decrease when porous structures are introduced (Zhou et al., 2012). It is necessary to investigate the electrical resistance (ρ, nΩ/m) of Cu-HFMs before their application as membrane cathodes in EMBR. The resistivity vs. porosity curve of the Cu-HFMs prepared at four different temperatures were plotted and fitted in two models (Langlois and Coeuret, 1989; Liu et al., 1999), as shown in Figure 3B and Supplementary S1.6. The Cu-HFM with 72.0 % porosity exhibits the highest resistivity (309.8 nΩ/m) while the Cu-HFM with 19.2 % porosity brings the lowest (104.8 nΩ/m). 4 times higher resistivity was estimated for the Cu-HFM with zero porosity and dense HF surface (measured with mercury porosimeter) compared to the solid copper metal in these two models, which could be attributed to the retained finger-like porous structures in the middle of the HF walls at very high sintering temperature (>700°C). For the Cu-HFMs with low porosity, better copper particle joint in the hollow fibers would lead to greater metal conductivity (Ma et al., 2005; Tang et al., 2010). However, even the Cu-HFM with a porosity of 72.0% exhibited much lower resistivity than compact stainless steel (690 nΩ/m) (Lide, 1995).

The mechanical strength of membrane materials is one of the key factors determining the application and lifetime of the membranes. Figure 3C plots the porosity and bending strength of the Cu-HFM vs. the reductive sintering temperature. The porosity of Cu-HFMs rapidly decreased from 72.0 % at 400°C to 19.2% at 700°C. Since the variation of porosity and sintering temperature always exhibit the opposite trends (Michielsen et al., 2013), it could be expected that the higher sintering temperature always leads to the denser hollow fibers (Figures 2i–l). In contrary to the porosity evolution trend, the bending strength of Cu-HFMs increases with the sintering temperature, from 52 MPa at 400°C to 76 MPa at 500°C and then climbing to 124 MPa at 625°C. The different variation trends of the porosity and mechanical strength could be attributed to the loose structures within the highly porous fibers (Yang et al., 2008). However, it should be noted that the hollow fiber sample possessing high mechanical strength but no desirable porous structure is pointless to serve as MBR membranes, which rules out the possibility to utilize the copper hollow fibers reduced at 700°C as the EMBR cathode.

The permeability of the Cu-HFMs was evaluated by measuring their N_2_ gas permeance and water permeability at room temperature. The N_2_ permeance of the Cu-HFMs was plotted against the corresponding reductive-sintering temperature as shown in Figure 3C. The largest permeance value is still in the same order of magnitude as previously reported for the stainless steel hollow fiber (~2 × 10^−4^ mol/m^2^ s p) (Michielsen et al., 2013). As can be seen, the N_2_ permeance gradually changed from 1.23 × 10^−4^ to 9.11 × 10^−5^ mol/m^2^ s pa when the sintering temperature increased from 400°C to 500°C, and continued to decrease to 1.56 × 10^−5^ mol/m^2^ s pa for the sample sintered at 625°C, which is in line with the porosity variation of Cu-HFMs. However, a drastic decline in N_2_ permeance (1.19 × 10^−9^ mol/m^2^ s pa) was noticed for the hollow fibers sintered at 700°C, which was about 14,000 times lower compared to Cu-HFMs sintered at 625°C. Huge difference here could be mainly attributed to the rare porous structures on the surface of the Cu-HFM reduced at 700°C in Figure 2l. The pure water permeability exhibited a similar trend to N_2_ permeance as shown in Figure 3D. Cu-HFMs prepared at 400°C obtained the highest pure water permeability (~ 8913 LMH/bar) while a water flux of only 227 LMH/bar was achieved for the Cu-HFM sintered at 700°C, which was a sharp decrease from 5812 LMH/bar for the sample reduced at 625°C. Although the Cu-HFM is not completely gas-tight or water-tight, the low water flux suggests that the Cu-HFMs prepared at 700°C are not suitable for the water filtration application. Considering the temperature required for the complete removal of polymer binders, better electrical conductivity, good permeation and pore size uniformity, we decided to use Cu-HFMs reduced at 625°C for our EMBR studies.

**Figure 4:**
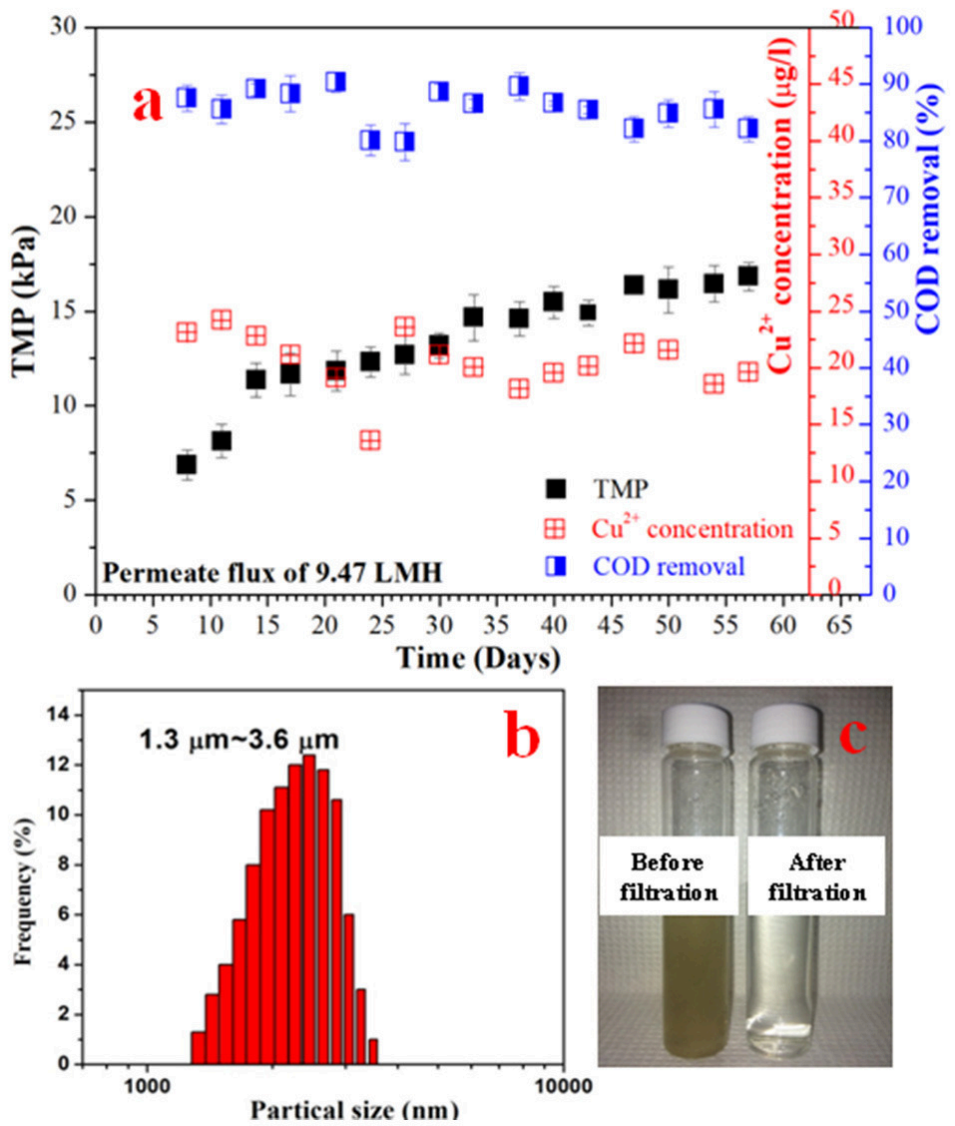
The TMP, COD removal rate and Cu^2+^ concentration for the EMBR system operated at 0.9 V **(a)**; **(b)** The solid average particle sizes of EMBR media before filtration; **(c)** The comparison effect of effluent before and after filtration.

In order to investigate their filtration and anti-biofouling performances, the Cu-HFMs were utilized in EMBR systems as cathode materials and microfiltration membranes. The EMBR reactor setup for wastewater reclamation was illustrated in Schematic 1 and detailed information about reactor construction and operation could be found in SI S1.5 and previous literature (Katuri et al., 2014; Werner et al., 2016). After initial cycles of bacterial enrichment, the EMBR with the Cu-HFM cathode produced an average current density of 2.79 ± 0.15 A m^−2^ at an applied voltage of 0.9 V, which was comparable to the data reported with the similar EMBR configuration, nickel hollow fiber cathode and same applied potential (Werner et al., 2016). The current density stabilized after 20 days of bacterial enrichment as shown in Figure S2, indicating the adaptation of suspended bacterial cells to the copper cathode in EMBR and the possible bacterial colonization on the carbon brush anode. After 57 days of the EMBR operation, the current density remained constant without any current leap or plunge, suggesting the stability of the whole system with the Cu-HFM cathode. To further illustrate the impacts of copper corrosion on the EMBR performances, the copper ion concentration in the EMBR media was monitored every 4 days during operation. From Figure 4a, it's quite clear that the copper concentration never exceeded 25 μg/L in the EMBR media, which exhibited the excellent anti-corrosion properties of the Cu-HFM cathode in EMBR system and was far less than the amount required for killing bacterial cells (Wu et al., 2010; Bian et al., 2018; Kimber et al., 2018). One point we must mention is that the synthetic wastewater used in our experiment contains copper ions (25.6 μg/L), which served as one trace element for bacterial growth on the anode and in the suspension for COD removal. The synthetic wastewater was chosen in this experiment because the same media has been demonstrated to be fine with bacterial growth in other EMBR systems (Werner et al., 2016). Also the copper concentration was much lower compared to the maximum copper concentration value (2 mg/L) for drinking water that the World Health Organization (WHO) recommends (WHO, 2004). Baudler et al. (Baudler et al., 2015, 2017) also observed little copper ions in their microbial electrochemical systems when they set a relatively negative potential on the copper electrodes, which was in line with our experimental results. The Cu-HFMs were thus proven to be capable of working normally in EMBR systems for wastewater treatment.

The COD concentration in the EMBR system was monitored after around 4 days in each fed-batch cycle and was found to decrease from 320 to 40.6 ± 6.81 mg L^−1^, which represented an average of 86.5 ± 2.27% COD removal efficiency as shown in Fig 4a. As there were no oxidizing substances dosed in the media, the organic removal could be attributed to the organics-oxidizing bacteria or anodic oxidation. However, in the abiotic study, we didn't notice too much changes regarding the COD concentration. So the anodic oxidation couldn't be the reason for COD removal. The carbon brush bio-anode was thus believed to efficiently remove the COD and exhibit the superior performances of the EMBR system for wastewater treatment similar to other BES systems (Cusick et al., 2012; Escapa et al., 2016).

As for the microfiltration function of the Cu-HFM cathode, we first analyzed the particle size ranging from 1.3 μm to 3.6 μm in the media after 4 days' feeding into EMBR, as illustrated in Figure 4b, which is far larger than the pore size of the Cu-HFM cathode sintered at 625°C. The particles in the EMBR media mainly consists of bacterial cells, extracellular polymeric substances (EPS) and suspended solids (Ren et al., 2014). The effluent filtered through the Cu-HFM cathode at the end of each batch exhibited excellent transparency compared to the media in the EMBR system, producing high-quality permeate with low TSS (~11 mg/L). To further characterize the permeate quality, UV-Vis absorbance spectrometry at wavelength 600 nm was utilized to measure the bacterial cell density and the media turbidity before and after Cu-HFM filtration, which is a common practice to analyze the effluent quality before and after wastewater treatment (Katal and Pahlavanzadeh, 2011; Hassanshahian et al., 2013). The UV absorbance decreased from 0.09–0.12 to 0.007–0.013 cm^−1^ after Cu-HFM filtration, which indicated significant reduction of suspended solids and bacterial cells in the filtered permeate and was comparable to the permeate quality (0.006–0.010 cm^−1^) from MBR waste treatment plant in KAUST using 0.4 μm polymer membranes. This clearly illustrated the superior biomass rejection of the Cu-HFM cathode as well (Figure 4c). Also, the membrane biofouling wasn't a severe issue as we had expected. Katuri et al. (Katuri et al., 2014) reported a TMP of more than 50 kPa with a permeate flux of 6.9 LMH after about 60 days of anaerobic EMBR operation using nickel hollow fiber membranes (Ni-HFM) as cathodes, which mainly resulted from the thick biofilm formation on the Ni-HFM cathode surface. However, in this study, the TMP increased from 6.86 ± 0.78 kPa (Day 8) to 16.82 ± 0.74 kPa at a flux rate of 9.47 LMH after more than 57 days of EMBR operation with the Cu-HFM cathode (Figure 4a), which represented a >80% TMP reduction with more permeate production and indicated the far less biofouling on the Cu-HFM cathode. This could help save massive energy required for EMBR systems and be probably attributed to the excellent anti-bacterial properties of the Cu electrode compared to Ni.

**Figure 5:**
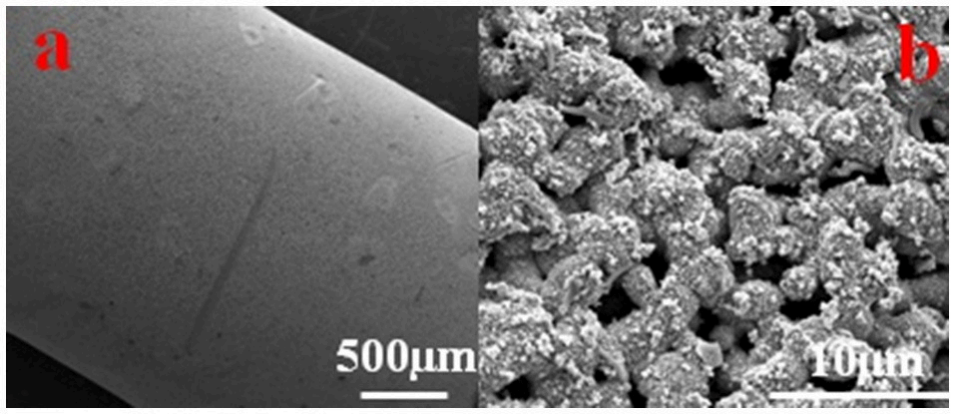
SEM images of Cu-HFM cathode after 57d of operation. scale size of **(a)** 500 μm and **(b)** 10 μm.

SEM images confirmed the hypothesis we made above, as no continuous biofilm formation was observed except some isolated bacterial cells, EPS and inorganic particles. The surface morphology of the Cu-HFM cathode in Figure 5 clearly exhibited the huge difference from Ni-HFM electrodes, where a much thicker biofilm (~4 μm) was depicted containing larger quantity of EPS and bacterial cell networks (Katuri et al., 2014; Werner et al., 2016). From Figure 5b, the well-preserved porous structures were clearly illustrated on the Cu-HFM cathode without any blockage by the biofilms or suspended solids. This visually confirms the anti-bacterial properties of the Cu-HFM cathode, which resulted in far less biofouling and much lower TMP values even after 57 days of operation compared to the data reported in literature (Katuri et al., 2016; Werner et al., 2016). Although Baudler et al. (Baudler et al., 2015, 2017) observed the thick biofilm formation on the copper anodes in microbial fuel cells (MFCs), Myung et al. (Myung et al., 2018) reported that less biofilm and proteins existed on the copper mesh cathode in the MFC, preventing the long-term biofouling in BES system. This suggests different BES performances when copper serves as cathodes and anodes, where different bacterial species colonize on the electrode surface. Further EDS analysis of the Cu-HFM cathode surface indicated copper to be the dominant surface element with more than 70 wt% after 57 days of EMBR operation, as shown in Figure S3. Low content of carbon (6.23 wt%), oxygen (13.75 wt%) and calcium (3.94 wt%) elements demonstrated the poor surface coverage of bacterial cells and solids, which reflected the excellent anti-fouling properties of the Cu-HFM cathode from another aspect. The EMBR system with Cu-HFM cathode was thus thought to be reliable for biofouling control and microfiltration.

## Conclusion

In this study, highly conductive Cu-HFMs (resistivity of 104.8 to 309.8 nΩ·m) were successfully prepared from copper powders via a phase-inversion process, combined with oxidation-reduction sintering technology. The micro-structures and properties of the resultant Cu-HFMs are highly dependent on the reductive-sintering temperature. In order to remove the polymer binder, obtain the desired porous structure (~1 μm) and appropriate mechanical strength (124 MPa), sintering and reduction of the Cu-HFM precursors were performed at the temperatures above 600°C, achieving excellent nitrogen permeance of 1.56 × 10^−5^ mol/m^2^ s pa and high pure water permeability of 5812 LMH/bar.

The Cu-HFMs were applied as the conductive cathodes, as well as MF membranes, in the EMBR system and obtained excellent permeate quality, biomass rejection and COD removal efficiency. Little biofouling and copper corrosion were found, suggesting the superior antimicrobial properties of copper membrane cathode in this system. Through the uses of Cu-HFMs as filter membrane and cathode in the EMBR system, massive energy could be saved via the fouling control in wastewater filtration and scale-up of EMBRs with Cu-HFM cathodes may be a feasible option for wastewater treatment in the near future.

## Author contributions

DL and BB conducted the experiment and wrote the manuscript. XC and ZL played active roles in analysing the results and provided constructive suggestions. BB and YS did major work of paper revision and finalization.

### Conflict of interest statement

The authors declare that the research was conducted in the absence of any commercial or financial relationships that could be construed as a potential conflict of interest.
