# Supplementary material for: Dual-Function Conductive Copper Hollow Fibers for Microfiltration and Anti-biofouling in Electrochemical Membrane Bioreactors
Source: Front Chem. 2018 Sep 25;6:445. doi: 10.3389/fchem.2018.00445 (PMC6167433; doi:10.3389/fchem.2018.00445)
Supplement: Supplementary file 1 [file Table_1.docx]

*Supplementary Information for*

Dual-function Conductive Copper Hollow Fibers for Microfiltration and Anti-biofouling in Electrochemical Membrane Bioreactors

Defei Liu^1, 2, 3^, Xin Chen^1, 2^, Bin Bian^3*^, Zhiping Lai^3^, Yue Situ^2*^

^1^ School of Environment and Chemical Engineering, Foshan University, Foshan 528000, China

^2^ School of Chemistry and Chemical Engineering, South China University of Technology, Guangzhou 510641, China

^3^ Department of Environmental Science and Engineering, King Abdullah University of Science and Technology, Thuwal 23955-6900, Kingdom of Saudi Arabia

**Table S1 List of abbreviation**

| *Common name* | *Abbreviation* |
| --- | --- |
| Membrane bioreactors | MBRs |
| Microfiltration | MF |
| Cu hollow fiber membranes | Cu-HFMs |
| Electrochemical membrane bioreactor | EMBR |
| Total suspended solids | TSS |
| Transmembrane pressure | TMP |
| Bio-electrochemical system | BES |
| Chemical Oxygen Demand | COD |
| Hollow fiber precursors | HFPs |
| Extracellular polymeric substances | EPS |

**S1 Experimental details**

**S1.1 Materials**

Cu particles (Particle size ~ 1 μm) was purchased from Hongxin material technology CO., LTD., China., NMP (N-methylpyrrolidone, AR grade, 99.0 %, Alfa Aesar) and PVP (polyvinylpyrrolidone, MW~36,000, Sigma) were used as solvent and additive, the polymer binder, Polysulfone (Ultrason E6020P) were purchased from BASF, respectively. All the chemicals were used as received without further puriﬁcation.

**S1.2 Preparation of Cu dope solution.**

To prepare the dope solution, PSF and PVP were added into N-methylpyrrolidone followed by stirring for 4 h. Cu particles were then added into the above solution. The mixed solution was stirred for 10 h until all Cu particles were completely dispersed into the solution and form a uniform suspension. The contents of PSF, NMP, Cu particles and PVP in the dope solution were controlled at 8.0, 27.0, 64.0 and 1.0 wt%, respectively.

**S1.3 Fabrication of Cu-HFMs precursors**

The Cu-HFMs precursors were fabricated from above dope solution via a phase inversion spinning process. Before spinning, the dope solution was degassed under vacuum for 3 h and leaving the dope solution for 12 h in dry air. The dope solution was extruded by a syringe pump through a spinneret (diameter of Din and Dout are 1.25 and 2.35 mm) into water for exchanging the NMP. Deionized water was pumped through the inner bore of the spinneret in a speed of 7.1 cc/min. The Cu-HFMs precursors were passed through a water bath for 12 h to complete the solidification process and thoroughly washed in water. Then the precursors were dried at room temperature for 8 h. The details of the procedure on HFMs spinning have been described in elsewhere.(Wang and Lai, 2012).

**S1.4 Oxidizing-reductive sintering of Cu-HFMs**

The Cu-HFM precursors were sintered at 600 ℃ for 3.5 h (heating rate: 5 ℃ min^-1^ and cooling rates: 5 ℃ min^-1^) in static air atmosphere to remove the polymer binders. The Cu-HFMs precursors were oxidized as CuO-HFM during this sintering process. Then the CuO-HFM was reduced from the metal oxide state to the metal state and sintered at 400 ℃, 500 ℃, 625 ℃, and 700 ℃, respectively for 6 h (ramping rate of 3 ℃ min^-1^ for heating and cooling down) in hydrogen/argon mixture gases flow of 150 cc min^-1^ (volume ratio of 3:7).

**S1.5 EMBR construction and operation details**

The single-chamber EMBR (250 ml volume) with 3 ports was constructed with Cu-HFM cathodes (projected surface area of 4.43 cm^2^) prepared above. All the reactors were operated in duplicates, equipped with carbon fiber brush anodes (8 cm × 3 cm, ZOLTEK) wired by two titanium rods, Cu-HFM cathodes and Ag/AgCl reference electrodes inserted from 3 ports, respectively. One end of the Cu-HFM cathode was connected to a peristaltic pump (Masterflex L/S, Cole-Parmer) and sealed with epoxy. Both the anode and the cathode were submerged in 250 ml of synthetic wastewater with acetate (0.32 g COD/L) described by Katuri et al (Katuri et al., 2014) and inoculated with anoxic digest sludge (10% v/v, KAUST Wastewater Treatment Plant, Thuwal, KSA). A voltage of 0.9 V was applied to EMBRs using a power source (3645A, Circuit Specialists) to prevent the possible copper corrosion and accumulate bacteria biofilm on the carbon brush anode for COD removal. A 10-Ω resistor was connected in the circuit and the voltage across the resistor was recorded by a data logger (ADC 24, PicoLog). After bacterial enrichment for one month and the peak voltage reached steady state, no sludge was added to the EMBR chamber except the synthetic wastewater containing 320 mg COD/L. Before we changed the batch at 20% peak current density in each batch, the COD concentration was detected with Hach COD kit to characterize the EMBR COD removal efficiency, as well as the transmembrane pressure (TMP) detected by a pressure sensor (68075-32, Cole-Parmer) when ~50% of the treated media was filtered through HFM cathodes at a permeate flux of 9.47 L/m^2^/h (LMH) at the end of each batch.(Katuri et al., 2014;Werner et al., 2016) The copper ion concentration was analyzed with ICP-MS (7500 Series, Agilent Technologies) to identify whether copper corrosion occurred during EMBR operation. The particle size in the EMBR media was detected by a laser particle analyzer (Zetasizer, Malvern). To further characterize the permeate quality, UV-Vis spectrometry at wavelength 600 nm was utilized to measure the media absorbance before and after Cu-HFM filtration, which is a common practice to analyze the effluent quality before and after wastewater treatment (Katal and Pahlavanzadeh, 2011;Hassanshahian et al., 2013). All the EMBRs were operated in anaerobic conditions for more than 50 days.

**S1.6 Resistivity modeling**

So far, several models have been proposed to bridge the relationship between resistivity and porosity for porous metals. For instance, after measuring the electrical conductivity of Ni foam, Langlois and Coeuret (Langlois and Coeuret, 1989) firstly proposed a half-empirical formula relating the resistivity and the porosity for porous material with high porosity as follows:

$\rho=\frac{4}{(1-\varepsilon)}\rho_{0}$…………………………..………………. Eq. (1)

where ρ and ρ_0_ are the resistivity of the porous and solid metals, respectively, and ε stands for the porosity. Eq. (1) is obtained from only a few experiments based on the nickel foams. Taking the differences of other metal materials into account, the formula was revised to be Eq. (2).(Liu et al., 1999)

$\rho=K\frac{4}{(1-\varepsilon)}\rho_{0}$……………………………………….Eq. (2)

Where K is the coefficient dependent on manufacturing technology, or more precisely, on the specific structure of porous metals. In this work, by fitting with the experimental data, it was found that the K value was equal to 1.22.

**Supplementary Figures:**


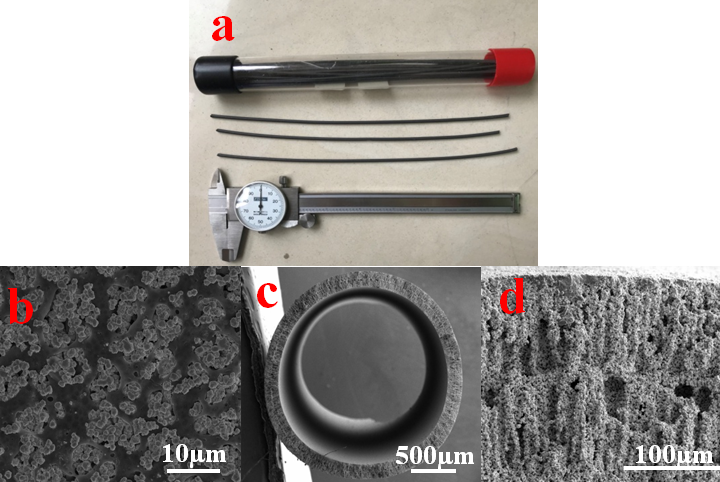


**Fig. S1 (a) The photograph and (b) SEM picture (surface) of Cu-HFM precursors; (c) and (d) the cross-sectional SEM picture of CuO-HF.**

**
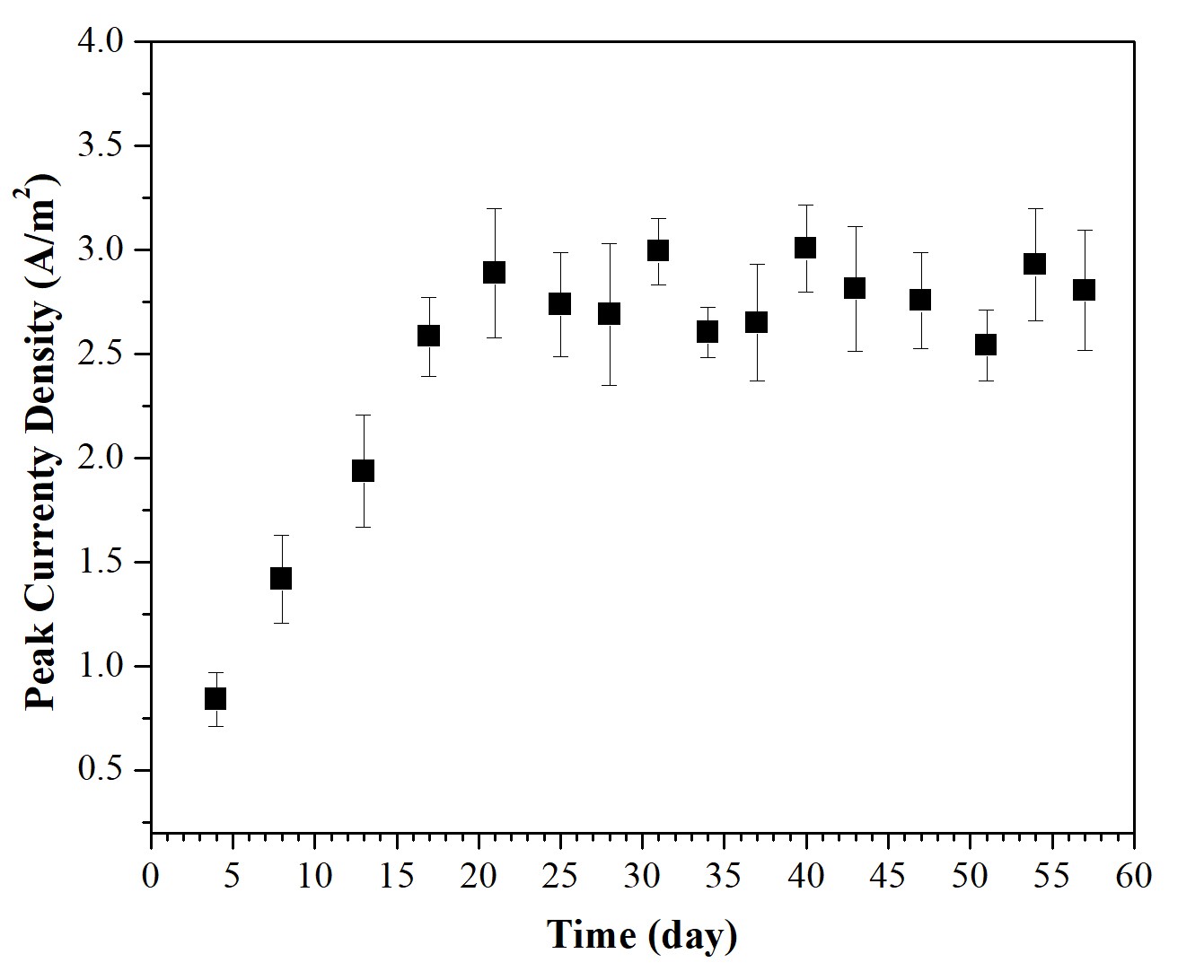
**

**Fig. S2 Peak current density of Cu-HFM cathodes in the EMBR at an applied potential of 0.9 V.**

**
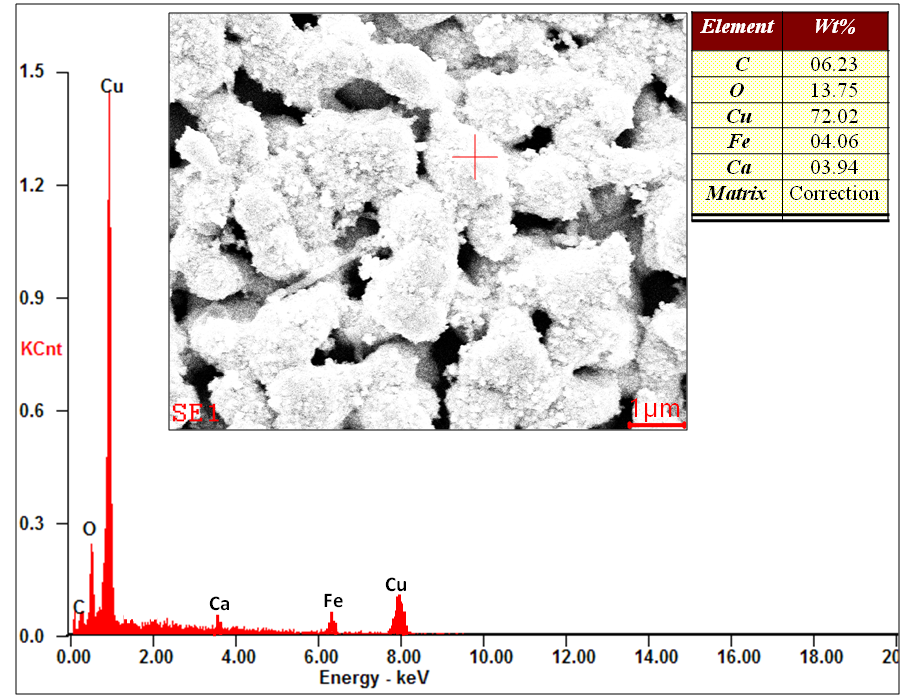
**

**Fig. S3 Energy Dispersive X-Ray Spectroscopy (EDS) analysis of the Cu-HFM cathode surface in EMBR after 57 days of operation.**

**REFERENCES**

Hassanshahian, M., Ahmadinejad, M., Tebyanian, H., and Kariminik, A. (2013). Isolation and characterization of alkane degrading bacteria from petroleum reservoir waste water in Iran (Kerman and Tehran provenances). *Marine Pollution Bulletin* 73**,** 300-305.

Katal, R., and Pahlavanzadeh, H. (2011). Influence of different combinations of aluminum and iron electrode on electrocoagulation efficiency: Application to the treatment of paper mill wastewater. *Desalination* 265**,** 199-205.

Katuri, K.P., Werner, C.M., Jimenez-Sandoval, R.J., Chen, W., Jeon, S., Logan, B.E., Lai, Z.P., Arny, G.L., and Saikaly, P.E. (2014). A Novel Anaerobic Electrochemical Membrane Bioreactor (AnEMBR) with Conductive Hollow-fiber Membrane for Treatment of Low-Organic Strength Solutions. *Environmental Science & Technology* 48**,** 12833-12841.

Langlois, S., and Coeuret, F. (1989). Flow-through and Flow-by Porous-Electrodes of Nickel Foam .1. Material Characterization. *Journal of Applied Electrochemistry* 19**,** 43-50.

Liu, P.S., Li, T.F., and Fu, C. (1999). Relationship between electrical resistivity and porosity for porous metals. *Materials Science and Engineering a-Structural Materials Properties Microstructure and Processing* 268**,** 208-215.

Wang, B., and Lai, Z.P. (2012). Finger-like voids induced by viscous fingering during phase inversion of alumina/PES/NMP suspensions. *Journal of Membrane Science* 405**,** 275-283.

Werner, C.M., Katuri, K.P., Hari, A.R., Chen, W., Lai, Z.P., Logan, B.E., Amy, G.L., and Saikaly, P.E. (2016). Graphene-Coated Hollow Fiber Membrane as the Cathode in Anaerobic Electrochemical Membrane Bioreactors - Effect of Configuration and Applied Voltage on Performance and Membrane Fouling. *Environmental Science & Technology* 50**,** 4439-4447.
